# Connectivity and Dispersal Patterns of Protected Biogenic Reefs: Implications for the Conservation of *Modiolus modiolus* (L.) in the Irish Sea

**DOI:** 10.1371/journal.pone.0143337

**Published:** 2015-12-01

**Authors:** Kate Gormley, Clara Mackenzie, Peter Robins, Ilaria Coscia, Andrew Cassidy, Jenny James, Angela Hull, Stuart Piertney, William Sanderson, Joanne Porter

**Affiliations:** 1 School of Life Sciences, Heriot-Watt University, Riccarton, Edinburgh, EH14 4AS; 2 Laboratory of Biodiversity and Evolutionary Genomics, University of Leuven, Ch. Deberiotstraat, 32, 3000 Leuven, Belgium; 3 Medical Research Institute, University of Dundee, Human Genetics Unit, Ninewells Hospital and Medical School, Dundee, DD1 9SY; 4 School of the Built Environment, Heriot-Watt University, Riccarton, Edinburgh, EH14 4AS; 5 School of Ocean Sciences, Bangor University, Menai Bridge, LL59 5AB; 6 The Institute of Biological and Environmental Sciences, Zoology Building, Tillydrone Avenue, Aberdeen, AB24 2TZ; National Cheng-Kung University, TAIWAN

## Abstract

Biogenic reefs created by *Modiolus modiolus* (Linnaeus, 1758) (horse mussel reefs) are marine habitats which support high levels of species biodiversity and provide valuable ecosystem services. Currently, *M*. *modiolus* reefs are listed as a threatened and/or declining species and habitat in all OSPAR regions and thus are highlighted as a conservation priority under the EU Marine Strategy Framework Directive (MSFD). Determining patterns of larval dispersal and genetic connectivity of remaining horse mussel populations can inform management efforts and is a critical component of effective marine spatial planning (MSP). Larval dispersal patterns and genetic structure were determined for several *M*. *modiolus* bed populations in the Irish Sea including those in Wales (North Pen Llŷn), Isle of Man (Point of Ayre) and Northern Ireland (Ards Peninsula and Strangford Lough). Simulations of larval dispersal suggested extant connectivity between populations within the Irish Sea. Results from the genetic analysis carried out using newly developed microsatellite DNA markers were consistent with those of the biophysical model. Results indicated moderately significant differentiation between the Northern Ireland populations and those in the Isle of Man and Wales. Simulations of larval dispersal over a 30 day pelagic larval duration (PLD) suggest that connectivity over a spatial scale of 150km is possible between some source and sink populations. However, it appears unlikely that larvae from Northern Ireland will connect directly with sites on the Llŷn or Isle of Man. It also appears unlikely that larvae from the Llŷn connect directly to any of the other sites. Taken together the data establishes a baseline for underpinning management and conservation of these important and threatened marine habitats in the southern part of the known range.

## Introduction

**Fig 1:**
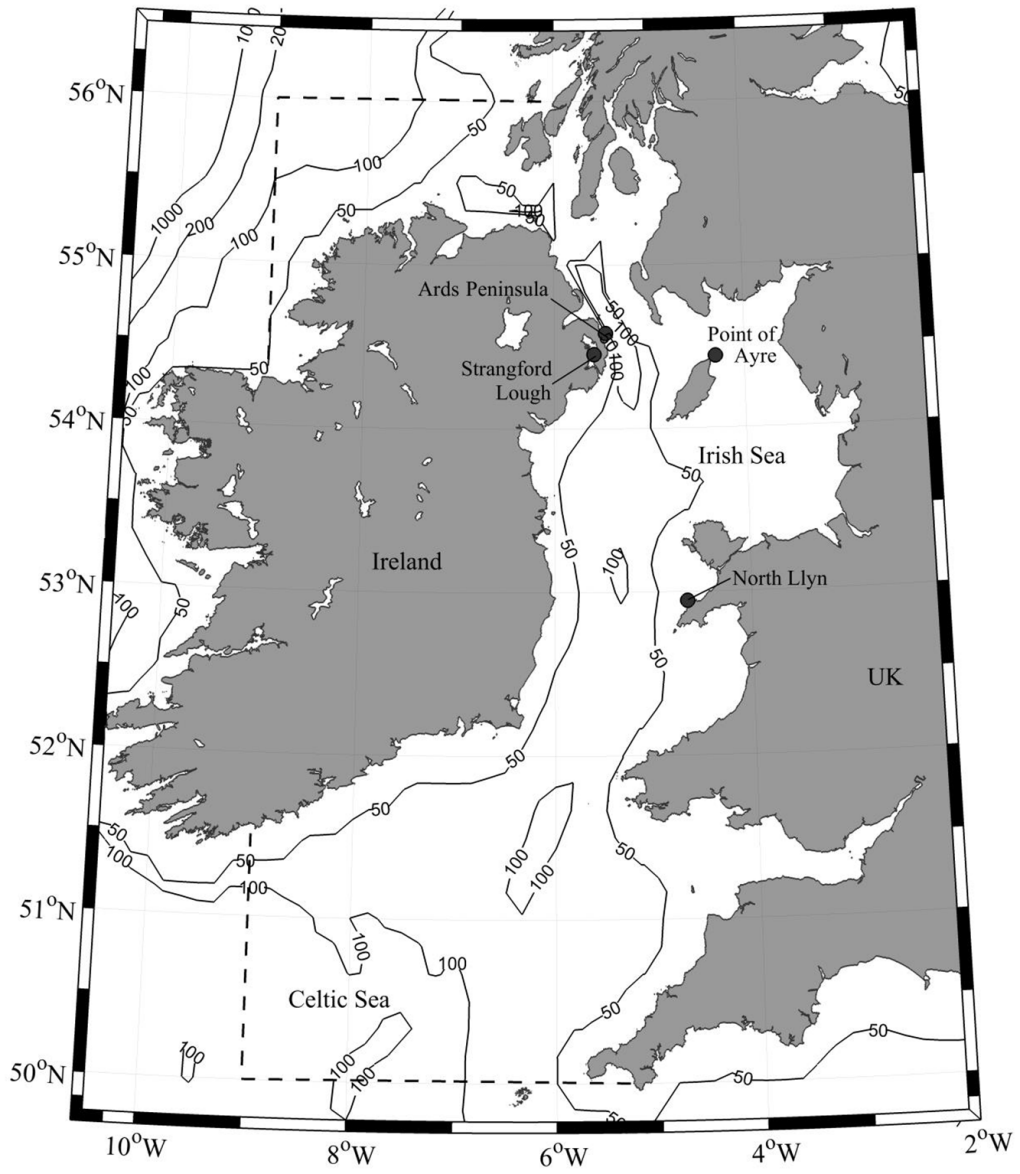
A bathymetric map of the Irish Sea, showing the four sample sites (black circles) within the model domain (indicated by the dashed line).

The marine bivalve *Modiolus modiolus* (Linnaeus, 1758) (horse mussel) is an Arctic-Boreal species with a distribution that extends from the seas around Scandinavia and Iceland southward to the Bay of Biscay [1]. *M*. *modiolus* reefs are considered a type of Annex I biogenic reef under the Habitats Directive (Council Directive 92/43/EEC on the conservation of natural habitats and of wild fauna and flora) [2]. These reefs are scarce and limited in their distribution in contrast to records of individuals and comprise dense continuous beds, or scattered aggregations of this large mussel [2]. Existing data places the southern limit of these reefs in the Irish Sea [1] where well-documented locations include the Ards Peninsula and Strangford Lough (Northern Ireland), the Point of Ayre (Isle of Man), and the North Llŷn (Wales) [3] (Fig 1). Horse mussel reefs can build up as a result of accretion of shell and faecal deposits [4, 5] and are typically characterized by high species diversity [6–8].

Decline in the spatial extent of *M*. *modiolus* reefs has been reported across the European distribution of the species [3]. In the Irish Sea, historical fishing activity such as scallop trawling and dredging has caused widespread, long-term damage to reefs including those situated around the Isle of Man and Northern Ireland [3]. Consequently, *M*. *modiolus* reefs are listed as a threatened and/or declining habitat in all OSPAR (Oslo-Paris Convention: The Convention for the Protection of the Marine Environment of the North-East Atlantic 1992) regions [9] and thus are a conservation priority under the EU Marine Strategy Framework Directive (MSFD) [10] since the connection has been made between “special habitats” (including OSPAR Priority Marine Habitats, PMHs; determined as “threatened and/or declining” under the OSPAR Convention 1992) and the achievement of Good Environmental Status (GES) [11]. It is therefore to be expected that more than half the horse mussel reefs in the Irish Sea are found in Marine Protected Areas (Strangford Lough, Point of Ayre and North Llŷn).

Conservation of species or biogenic habitats requires detailed knowledge about their demographic structure and status; with genetic data contributing to this definition. This information may be of use in informing the successful maintenance of these populations through breeding programmes [12], restoration of habitats [13] and/or design and implementation of Marine Protected Areas (MPAs) or Regional Management Units [14]. In addition, a thorough understanding of population connectivity has become a key requirement for determining and defining threats to marine biodiversity and is crucial to the marine conservation and management process [15, 16].

In the marine environment, population connectivity is principally driven by pelagic larval stages and is therefore directly influenced by oceanographic processes such as tidal currents, residual currents, and wind-driven currents [17]. However, patterns of dispersal of pelagic larvae remain poorly understood due to the difficulty of obtaining direct measurements of movement and behaviour of these propagules. This is especially problematic in the marine environment where sessile or sedentary species (such as *M*. *modiolus*) have a bipartite life cycle, periodically producing planktonic propagules that are virtually impossible to track over large distances [18] using currently available technologies [16]. Although in relatively constrained regions such as lagoons or coral reefs, intensive larval sampling can offer some model validation e.g. [19]. In light of these challenges, biophysical modelling (coupling hydrodynamic and particle tracking models) has emerged as a useful tool for simulating larval dispersal across time and space [20].

### Marine Spatial Planning for Connectivity and Conservation

Currently, there is a requirement to establish an ecologically coherent network of protected areas (MPAs, Marine Conservation Zones, MCZs and Natura 2000 sites) under the UK Marine and Coastal Access Act 2009, the OSPAR Convention and the EU MSFD; and develop Ecosystem Based Management (EBM) tools [21]. One way in which population genetics complement ecological approaches to conservation is through the use of molecular techniques to supplement demographic and population studies [17]. For example, use of both genetic techniques and larval dispersal modelling in tandem is required to provide sufficient information regarding the connectivity status of a habitat. Weersing and Toonen [16] showed that average pelagic larval duration is poorly correlated with genetic structure and an effective strategy for the conservation of a particular species should, in part, be determined by information gathered on its genetic structure, especially the spatial distribution of genetic variability [17].

Connectivity between populations contributes to increased resilience of a species under changing environmental conditions (e.g. climate change) where more robust source populations may provide larvae to other depleted populations [22]. In this way, sink populations can enhance population persistence across a network of connected populations [22]. McLeod *et al*. [23] suggested that biological patterns of connectivity should be taken into account when designing MPA networks and facilitate mutual replenishment and recovery from disturbance, particularly under the context of climate change. In addition, they recommended that future connectivity patterns be modelled so that measures can be taken to protect areas and facilitate expansion and migration [23], also discussed by Gormley *et al*. [24].

Disruption to connectivity, e.g. habitat fragmentation or changes to current regimes may lead to a loss of the habitat, or lack of recruitment, threaten ecosystem integrity and functions [25] and result in potential inbreeding [18]. In addition, placement of industry zones that potentially alter oceanographic processes (such as marine renewable technologies) may ultimately influence the trajectory of propagules. Importantly, the establishment of small and scattered MPAs that do not consider population connectivity may have a limited effect on the protection of the marine environment [15].

In this study, microsatellite markers were developed and used to elucidate genetic diversity and genetic structure within and among four *M*. *modiolus* reefs from Northern Ireland, Isle of Man and Wales. The major objectives were: a) to predict dispersal of larval propagules in the Irish Sea for *M*. *modiolus* reefs through the use of biophysical modelling coupled with a larval dispersal model; b) to estimate the level of genetic variation in *M*. *modiolus* and assess the efficiency of microsatellite markers for studying this species; c) to detect whether genetic differentiation has occurred among populations and identify factors that influence population genetic structure in this species; and d) to discuss factors contributing to the maintenance of connectivity of the populations and provide information that may be useful for developing future conservation management for this PMH.

## Methods

### Larval Dispersal Modelling

Simulated Lagrangian dispersal of virtual particles, representing *M*. *modiolus* larvae from the Irish Sea populations (Ards Peninsula, Strangford Lough, Point of Ayre, North Llŷn; Table 1), was carried out using the methodology described in Coscia *et al*. [20]. The biophysical modelling involved the development and validation of a three-dimensional (3D) hydrodynamic model (sbPOM) [26] of the Irish Sea [27], in order to simulate realistic tidal and residual currents throughout the Irish Sea. The model cell size was 1/30° (longitude) by 1/60° (latitude), giving a resolution of approximately 1.85 km, and the model had 20 vertical terrain-following layers. The standard prognostic variables (e.g., velocity, temperature, salinity, and turbulence) are solved using finite-difference discretization on a staggered, orthogonal grid. Detailed information of the development and validation of the model has been reported by Robins *et al*. [27]. A typical year (1990) in terms of both wind-induced bed shear stress and sea surface heating was simulated for this study. The simulation was forced with six primary tidal constituents including the semidiurnal M_2_ (lunar) and S_2_ (solar) constituents, derived from an outer-nested model [28]. The source of synoptic meteorological fields was the European Centre for Medium-Range Weather Forecasts-Interim reanalysis [29], available every 3 h at a (global) grid resolution of 1.5°.

**Table 1:** *Modiolus modiolus* reefs populations sampled.

Site	Collection Date	Latitude	Longitude	Extent	Depth	No. Genetic Samples	Authority
**1: Northern Ireland—Ards Peninsula**	August 2011	-5.457°	54.582°	1km^2^	34m	83	Department of Environment Northern Ireland
**2: Northern Ireland—Strangford Lough**	April 2011	-5.594°	54.454°	Currently unknown	25m	57	Department of Environment Northern Ireland
**3: Isle of Man—Point of Ayre**	June 2013	-4.305°	54.439°	16km^2^	34m	31	Isle of Man Government
**4: North Wales—North Llŷn**	June 2010	-4.654°	52.944°	6km^2^	33m	50	Natural Resources Wales

A Lagrangian particle tracking model (PTM) was developed for this study to predict the likely larval dispersal of *M*. *modiolus*. Output from the hydrodynamic simulation (3D velocities and horizontal diffusion), temporally and spatially interpolated to the individual position of each particle, each time step, was used to drive the PTM, so that particles were iteratively dispersed due to the simulated three-dimensional advection and horizontal diffusion. As the dispersal of *M*. *modiolus* larvae is poorly understood, the pelagic larval duration (PLD) is not well defined. Schweinitz and Lutz [30] suggested that larvae remained in the water column for 30 days, therefore 30 days was chosen as the PLD within the PTM. The model simulated passive larval transport; that is, no vertical migration due to larval swimming. Again, this method was employed since the dispersal of *M*. *modiolus* larvae is poorly understood, and acts as a baseline study for dispersal—sensitivity to generalised swimming behaviours has been studied in the region e.g.[27, 31]. Cohorts of 10,000 larvae were released from the seabed at the four sample locations (representing a single point within the reef and corresponding to collection sites for genetic material), and at six times throughout the season, with start dates chosen on the 1st of each month (April to September). Each particle was then—tracked for the 30 day PLD. Therefore, a total of 240,000 individual larval trajectories were simulated. Larvae that encountered land, or the sea surface or the bed, were reflected back to their position in the previous PTM time step.

### Development of polymorphic microsatellite markers

Permission for the collection of *Modiolus modiolus* samples for this study was granted by the following agencies: Loch Creran and Scapa Flow, Scotland, Scottish Natural Heritage; Ards Penninsula and Strangford Lough, Northern Ireland, Department of Department of Environment Northern Ireland; Point of Ayre, Isle of Man, Isle of Man Government; and North Llŷn, Wales, Natural Resources Wales.

*M*. *modiolus* (n = 10) were collected by SCUBA divers from Loch Creran (West Scotland) and from Scapa Flow (Orkney). Adductor muscle tissue was dissected from selected individuals and stored at 4°C in 96–100% ethanol. Genomic DNA extraction (from approximately 25mg of tissue) was carried out using the QIAGEN DNeasy Blood and Tissue kit protocol [32]. Microsatellite isolation was carried out according to Zane *et al*. [33]. Following the identification of 196 positive clones, each was amplified by Polmerase Chain Reaction (PCR) using SP6 and T7 vector primers. The PCR reactions were prepared using Illustra PureTaq Ready-To-Go PCR beads in a final reaction volume of 25μl (50ng of starting genomic DNA) and run on a G-Storm (KAPA BIOSYSTEMS) thermocycler at 94°C for 5 mins followed by 30 cycles of denaturation at 94°C for 30 s; annealing at 55°C for 30 s; elongation at 72°C for 30 s; and ending with a single step at 72°C for 5 mins. PCR reactions were run on a 1% agarose gel to check for a product, cleaned using an Invitrogen PureLink Quick PCR Purification kit and sent to the Genepool, Edinburgh for sequencing. The resulting data were analysed in Sequencher v 4.10.1 (GeneCodes Ltd) for the presence of microsatellite repeats. Primers were designed using the Websat portal [34]; minimum T_m_ 57–68°C; primer size 18-27bp; Primer GC 40–80%; maximum 3’ stability = 250 and maximum 3’ self-complementary = 2. Primer pairs that resulted in the amplification of a DNA fragment of the expected length, were taken forward for further testing under the following conditions after optimisation: DNA concentration of 20ng/ul at 94°C for 5 mins followed by 30 cycles of denaturation at 94°C for 30 s; annealing at a gradient temperature from 53°C to 58.1°C for 30 s; elongation at 72°C for 30 s; and ending with a single step at 72°C for 5 mins. Of the 34 loci initially identified, only 14 were considered to be suitable for further development. The forward primers for these loci were fluorescently labelled (Eurofins: FAM and HEX; and Applied Biosystems: NED). The optimal annealing temperature was 57°C for all loci, with DNA concentration 10- 20ng/ul. PCR fragments were run alongside a ROX500 size standard in an ABI 3130 Genetic Analyser and alleles were scored for peak height (base pairs, bp) using Genemapper version 3.5.

### Field Collection and screening of individuals from *Modiolus modiolus* reefs

Samples of *M*. *modiolus* for population screening were hand collected, between 2010 and 2013, by SCUBA divers from the reefs listed in Table 1 and illustrated in Fig 1.

DNA was extracted from all individuals as described previously and DNA quality was assessed via gel electrophoresis (1% agarose), samples showing poor quality were discarded. All individuals from the selected populations were genotyped using the 5 developed species-specific markers. PCR reactions were run as previously described with the optimised conditions.

Frequencies of null alleles were estimated using FreeNA [35]. Deviations of genotype frequencies from Hardy-Weinberg equilibrium (HWE) and linkage disequilibrium were tested in GENEPOP 4.0 [36]. Genetic variation was assessed through estimation of allelic frequencies, observed (H_o_) and expected heterozygosities (H_e_), F_is_ and F_st_ [θ estimator [37]] values. Allelic richness and number of private alleles were calculated using Fstat 2.9.3 [38] along with F_st_ pairwise test for differentiation (120 permutations).

Finally, population structure was analysed using two statistical approaches. Firstly, the Bayesian clustering techniques implemented in the software STRUCTURE 2.3.1 [39, 40]. STRUCTURE was run allowing for admixture and correlated allele frequencies using 500,000 iterations following a 100,000 burn-in period to ensure chain convergence using a LOCPRIOR model. STRUCTURE uses individual multilocus genotype data to cluster individuals into groups (k) while minimising Hardy-Weinberg disequilibrium. Δk is a good predictor for the real number of clusters in the data [40, 41]. The value of k was calculated from analysis of results in STRUCTURE Harvester [42] by averaging the mean posterior probability of the data L(k) over 5 independent runs. Post-processing of results was completed using CLUMPAK [43]. Secondly, the discriminant analysis of principal components (DAPC) was implemented in ADEGENET [44, 45] for R. This method maximises genetic separation among groups, whilst minimising variation within groups.

Finally, a connectivity matrix was constructed to show linkages between genetic spatial structure and biophysical model outputs. Seasonally-averaged and cohort-averaged connectivities between populations (percentages of the total 6 x 10,000 larvae) were overlaid with significant genetic differentiated populations. Criteria for settlement (connectivity/retention) were for larvae to be located within 10 km of the settlement site after 30 days simulation. Modelled larval release (source) sites are labelled on the y-axis, and settlement (sink) sites are located on the x-axis; genetic values do not identify source/sink sites.

## Results

### Larval Dispersal Modelling

**Fig 2:**
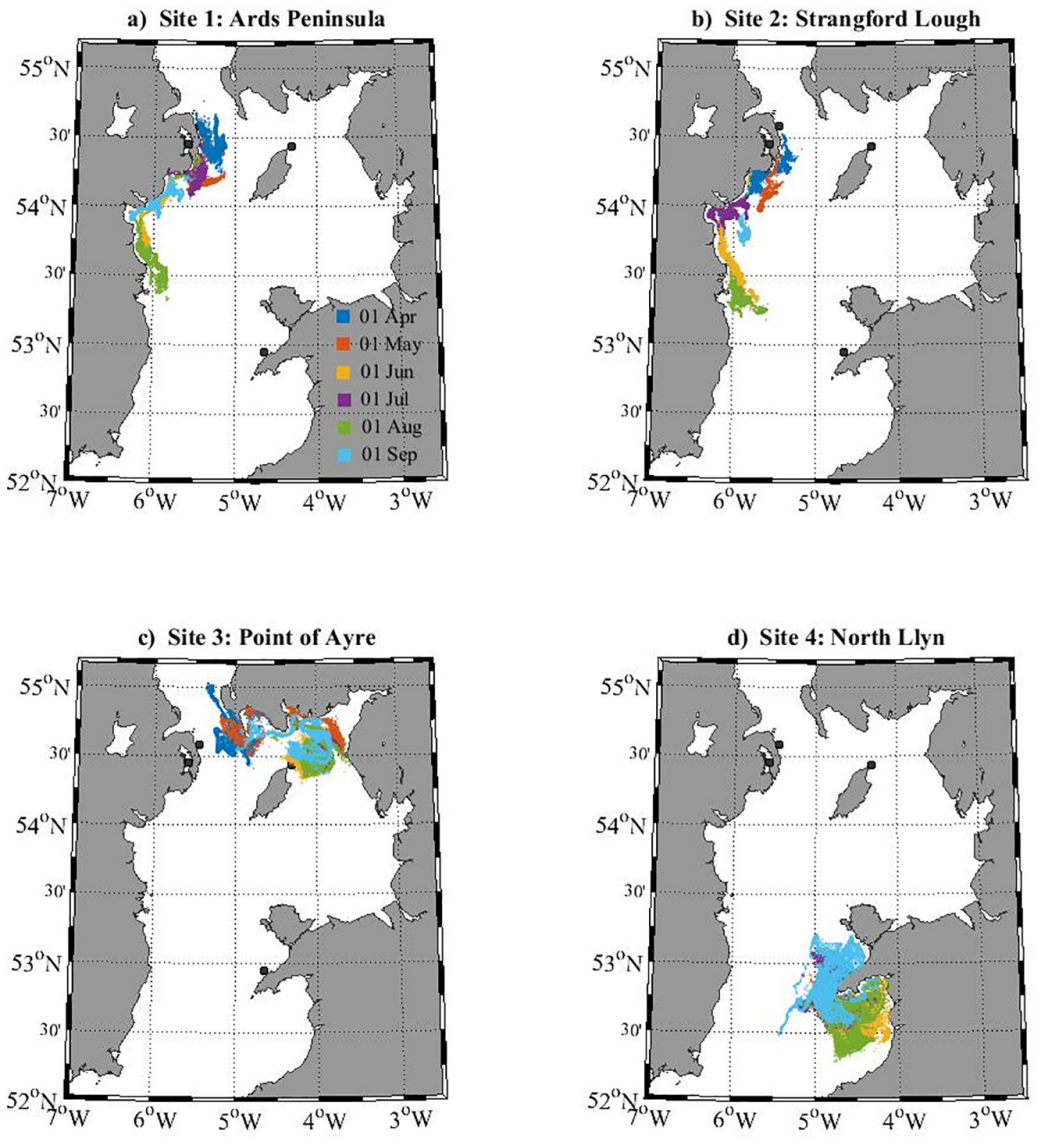
Larval dispersal maps for each simulated cohort of 10,000 larvae within the northern Irish Sea (see Fig 1). Each panel (a-d) displays PTM output, after a 30-day PLD, from six release dates (01 April—01 September), released from the distinct source locations (black circles).

**Fig 3:**
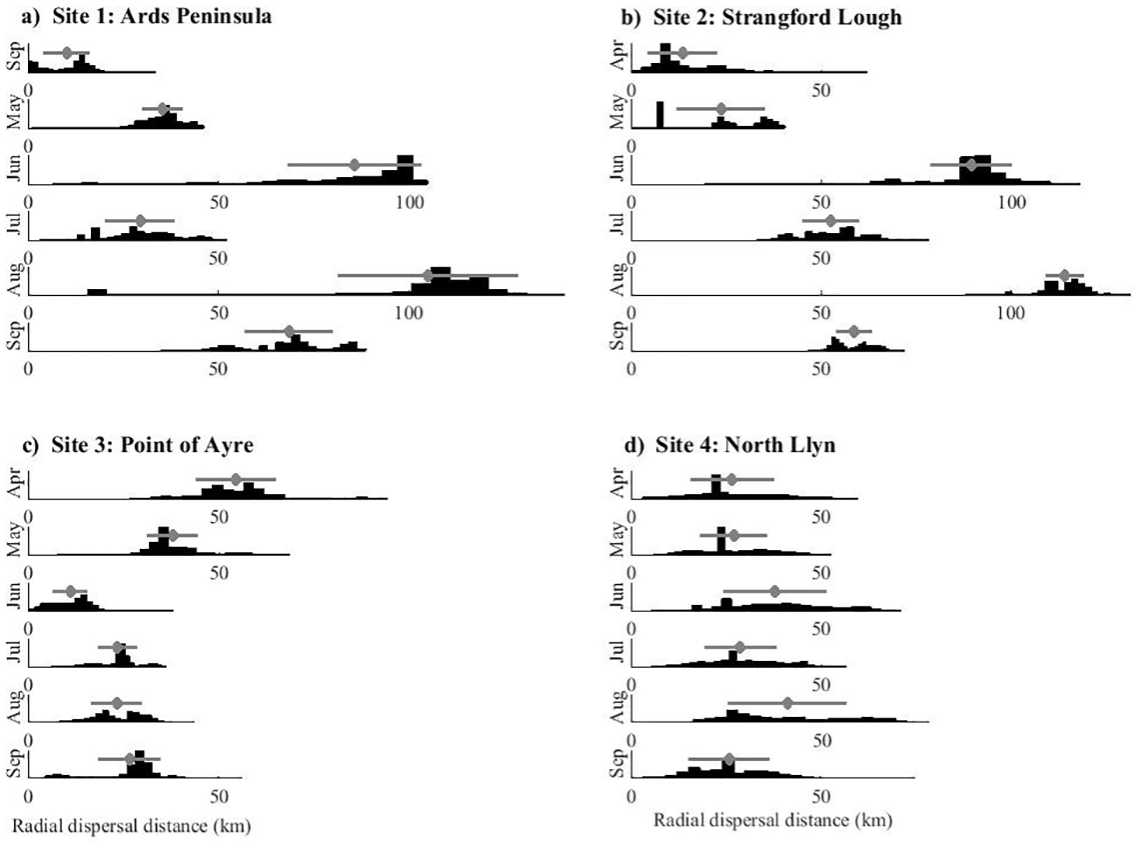
Histograms of radial dispersal distances for each simulated cohort of 10,000 larvae. Each Fig (a-d) displays histograms from six release dates (01 April—01 September), released from the distinct source locations (Sites 1–4). Each bar in each histogram represents 400 particles (i.e., each histogram is split into 25 bars). The average radial dispersal for each cohort is shown with grey diamonds and horizontal grey error bars signify one standard deviation. Radial dispersal distance is defined as the 30-day source-to-sink shortest distance.

The Particle Tracking Model simulated a general southerly movement of particles from the Northern Ireland release sites (Sites 1–2), over the 30-day PLD (Fig 2a and 2b), due to a strong southerly residual current in the western North Channel, in addition to the development of the cyclonic Western Irish Sea gyre [27, 46]. As the stratification strengthened during summer months, the simulated residual currents also strengthened [27], and larval dispersal distances generally increased (Figs 2 and 3). Point of Ayre (Isle of Man) populations (Site 3) were simulated to travel north towards Scotland, but not beyond the North Channel over the 30-day PLD (Fig 2c). For the North Llŷn site (Site 4), simulated larval transport was generally southerly over the 30 days, travelling around the head of the North Llŷn into the northern part of Cardigan Bay—larvae were simulated to travel slightly further south during June, July and August (when residual currents were strongest), than during April, May, and September over the 30 days (Fig 2d). A proportion of larvae each month, however, travelled north towards western Anglesey—more so during April and May when residual currents were weaker (Fig 2d). Seasonal variability in dispersal patterns was slightly less apparent for the North Llŷn and Point of Ayre sites (Fig 3), presumably because these sites were less influenced by strong residual currents, such as the Western Irish Sea gyre.

Although no connectivity was simulated between each release site, several simulations suggested that there could be some one-way connectivity. For example, particles released from the Point of Ayre (Isle of Man) during April were located within 15 km of Ards Peninsula after the 30-day PLD (Fig 3c). However, it appears unlikely that larvae from Northern Ireland will connect directly (i.e., during a 30-day PLD) with sites on the Llŷn, or the Isle of Man, under similar conditions to the simulated PTM. It also appears unlikely that larvae from the Llŷn will connect directly with sites in Northern Ireland, or the Isle of Man, over the 30-day PLD. Nevertheless, 30-day radial dispersal distances (i.e., direct source-to-sink dispersal distances) reached 150 km (From Ards north; Fig 3a), which is approximately the radial distance from Northern Ireland/Isle of Man sites to the Llŷn sites. Further, maximum radial dispersal distances were regularly greater than 60 km, which is approximately the radial distance from Northern Ireland sites to the Isle of Man site (Fig 3).

When looking at cohort-averaged radial dispersal distances (Fig 3), summer releases (particularly June and August) appear to be most ‘energetic’, and spring releases (April and May) least energetic. However, there is a high degree of variability, and the Point of Ayre site displays a different pattern of seasonal variability (Fig 3). This could be attributed to the (southwards and eastwards) residual currents opposing the (northwards and westwards) tidal residuals in that region [27].

### Development of polymorphic microsatellite markers

Table 2 shows the results of the 5 microsatellite loci characterised. The number of alleles ranged from 16 for Modimicro 13 to 70 for Modimicro 2. Observed heterozygosity and expected heterozygosity varied between 0.3458 for Modimicro 30 to 0.9480 for Modimicro 20; and 0.6276 (Modimicro 13) to 0.9635 (Modimicro 2), respectively.

**Table 2:** Characterisation of microsatellite loci in *Modiolus modiolus*.

Locus	Label	Repeat motif	Sequence (5' >- 3')	Base Pairs	Approximate size (bp)	T_a_ (°C)	N_a_	H_o_	H_e_
Modimicro 2 (F)	FAM	CA(30)	CTCCGCTATGTTTGACCATGTA	22	167–317	57	70	0.5072	0.9635
Modimicro 2 (R)			TCCACACCGAGTAACAAATCAG	22					
Modimicro 11 (F)	HEX	GAA(7)	AGAATCCTTTCTGTGTTGTCCG	22	163–349	57	22	0.4946	0.6743
Modimicro 11 (R)			CATCTGCCTACCTACAGTTCCC	22					
Modimicro 13 (F)	HEX	TC(11)	CACAGCCTCCTGGTCACAATA	21	150–200	57	16	0.4021	0.6276
Modimicro 13 (R)			TGGCGTGTTATTCTAGCAAATG	22					
Modimicro 20 (F)	NED	TCA(10)	AATTGCTCACTTGGCGTAAAAC	22	180–248	57	19	0.9480	0.7771
Modimicro 20 (R)			TGGAAATGGAGAGACAGATCCT	22					
Modimicro 30 (F)	FAM	CA(9)	CACACAAGACAGGCCAGATAGA	22	147–183	57	22	0.3458	0.7144
Modimicro 30 (R)			GAAGAATCCCCACAAACACATT	22					

### Population Screening for Genetic Connectivity

The average number of alleles (including all markers) per population ranged from 9.4 at Point of Ayre to 20.8 at Strangford Lough; and the number of private alleles was greatest at Strangford Lough (51) and least at Point of Ayre (31). The observed heterozygosity (H_o_) was lowest (0.4373) in the North Llŷn population and highest (0.5958) at Strangford Lough. Overall, the expected heterozygosity (H_e_) was higher than the observed heterozygosity indicating a possible heterozygote deficiency (Table 3).

**Table 3:** Genetic Diversity Parameters inferred from microsatellites.

Site	N	H_e_	H_o_	N_A_	A_R_	N_p_	F_is_	F_st_ (before and after ENA correction)			
								Ards Peninsula	Strangford Lough	North Llŷn	Point of Ayre
**Ards Peninsula**	50	0.8178	0.5432	18.20	13.23±7.86	16	0.338		0.00	0.05*	0.07*
**Strangford Lough**	51	0.8450	0.5958	20.80	14.65±7.54	31	0.297	0.01		0.07*	0.10*
**North Llŷn**	48	0.7186	0.4373	13.00	10.14±6.23	5	0.394	0.05	0.06		0.02
**Point of Ayre**	31	0.6241	0.5819	9.40	8.84±5.26	3	0.069	0.09	0.12	0.05	

N = number of samples; H_e_ = expected heterozygosity; H_o_ = observed heterozygosity; N_A_ = number of alleles; A_R_ = allelic richness; N_p_ = number of private alleles; F_is =_ inbreeding coefficient; F_st_ before (upper diagonal) and after (lower diagonal) ENA correction.

*Significant;

Indicative adjusted nominal level (5%) for multiple comparisons is: 0.0083 following Bonferroni correction, FStat

The presence of null alleles was detected for 4 of the 5 markers for the majority of the populations tested (r >0.05). Modimicro 20 locus showed no null alleles across all 4 populations and the Point of Ayre population reported very low (0.0001) null allele frequencies for loci Modimicro 11 and 30. Significant positive F_is_ values for all populations indicate a deficiency of heterozygotes compared to that expected under the Hardy-Weinberg Equilibrium (HWE). Therefore it was concluded that all populations were not in Hardy-Weinberg Equilibrium.

It has been demonstrated that the inclusion of markers with null alleles can lead to an overestimation of the genetic differentiation (F_st_). Given that all but one of the markers and populations contained null alleles, it was therefore not possible to exclude the null allele markers from the calculations. To allow for this, Estimation of Null Allele (ENA) correction was performed using FreeNA software and pairwise F_st_ was calculated before and after the correction procedure was applied [35]. Pairwise F_st_ values before ENA correction ranged from 0.0023 (Strangford Lough/Ards Peninsula) to 0.10 (Strangford Lough/Point of Ayre); and after correction ranged from 0.01 (Strangford Lough/Ards Peninsula) to 0.12 (Strangford Lough/Point of Ayre) (Table 3). Following correction, values remained in the same order of magnitude, indicating that the presence of null alleles was not overly influencing F_st_ estimates.

The Fstat pairwise test for differentiation showed low but significant (p<0.05) differentiation of Northern Ireland *M*. *modiolus* populations from Irish Sea counterparts (Ards Peninsula-Point of Ayre = 0.07; Ards Peninsula-North Llŷn = 0.05; Strangford Lough-Point of Ayre = 0.10; Strangford Lough-North Llŷn = 0.07). The Strangford Lough/Ards Peninsula and North Llŷn/Point of Ayre populations were not significantly differentiated.

**Fig 4:**
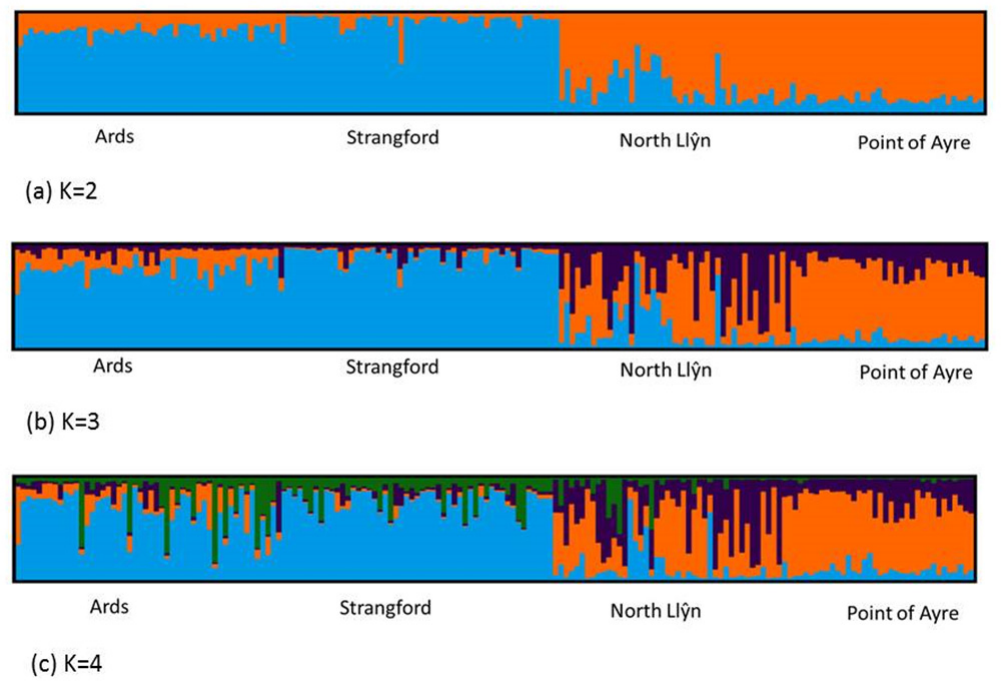
Structure plots for K = 2, K = 3 and K = 4.

**Fig 5:**
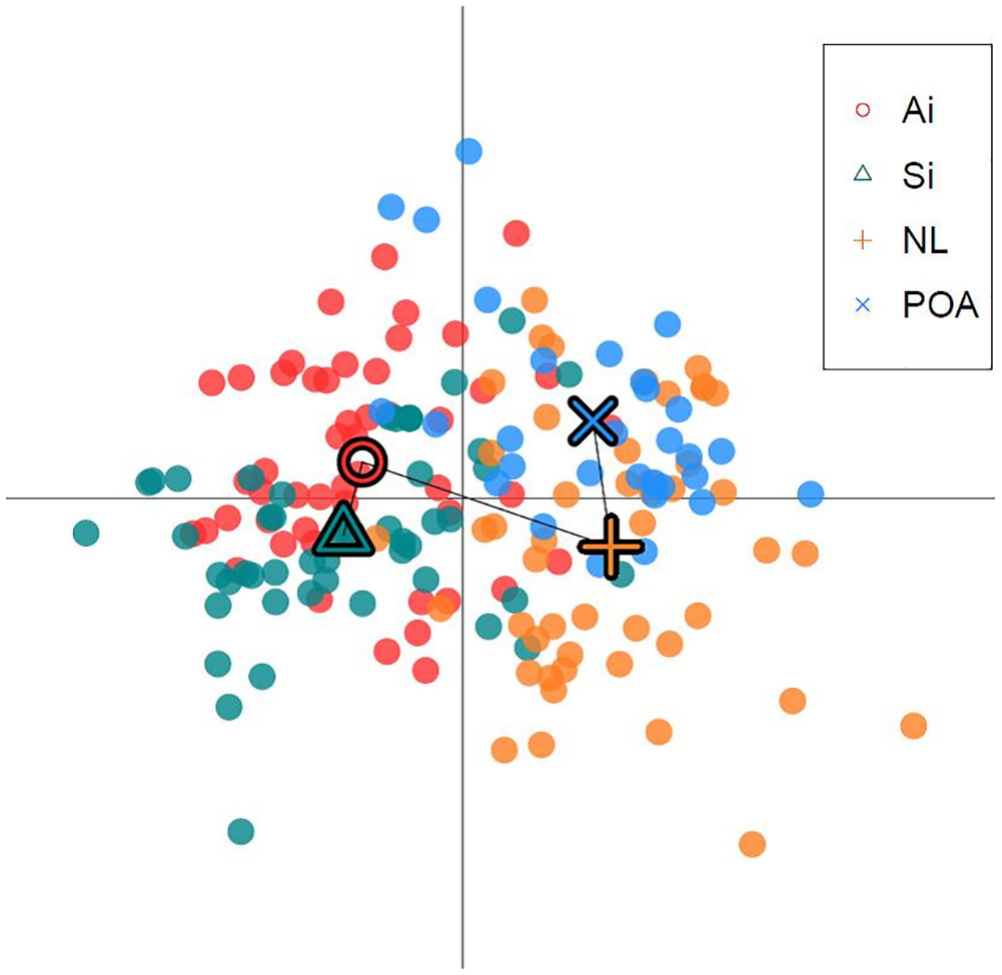
Plot of the DAPC analysis for groups defined by sampling location. Dots represent individuals and the centre of the population indicated by a corresponding shape, connected by a minimum *spanning tree*.

Results from data analysis in the software STRUCTURE and STRUCTURE Harvester indicated a mean Log Likelihood of K = 2 and the ‘Evanno method’ (the second order rate of change in the likelihood, DeltaK) K = 2 (Fig 4). The global Fst value (using ENA) was 0.06 (p<0.001), therefore indicating moderate genetic differentiation between populations as defined by Wright [47] and Balloux and Lugon-Moulin [48]. It was concluded that the Bayesian STRUCTURE clustering approach using multilocus microsatellite genotypes detected population structure; and the symmetric proportion of individuals assigned to putative clusters indicated genetic structure. The presence of population structure was confirmed by comparable results obtained within the DAPC analysis, with strong evidence for the presence of two genetic clusters (Fig 5).

**Fig 6:**
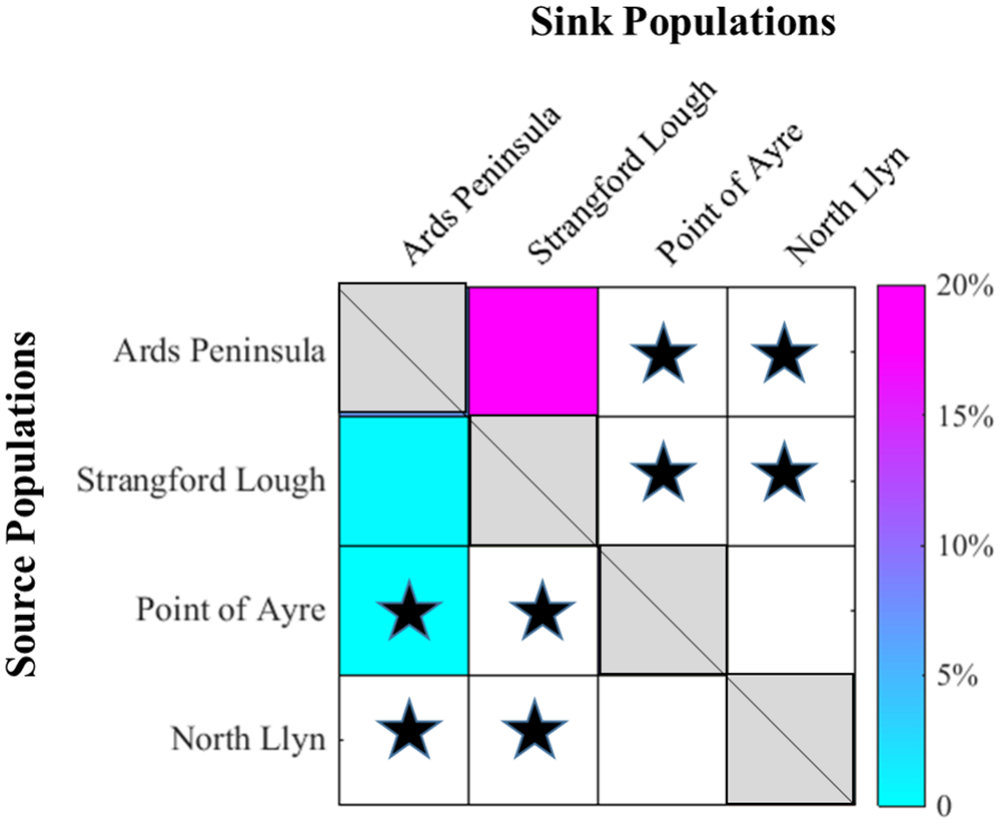
Connectivity matrix aligning larval dispersal and genetic differentiation. Blacks stars represent significant differentiation. Diagonal cells signify retention at the release sites, and coloured cells elsewhere signify directional connectivity. White cells signify no connectivity.

The connectivity matrix (Fig 6) from modelled results indicates that Strangford Lough is a sink site for larvae dispersed from the Ards Peninsula (20%), whereas, there is weaker flow of larvae in the opposite direction (<5%). The results also indicate that Point of Ayre is a potential source site for Ards (<5%), however there is no connectivity in the opposite direction. The modelled larval dispersal shows that there is no connectivity between the Ards/Strangford and the North Llŷn/Point of Ayre populations which is also indicated by the genetic differentiation results and the two population clusters shown by the STRUCTURE analysis. While, the modelled data showed no connectivity between Point of Ayre and North Llŷn populations, there was no genetic differentiation between these sites.

## Discussion

The combined approach of using genetic diversity markers with population dispersal modelling have, in the past, not been fully incorporated into the design and placement of Marine Protected Areas (MPAs) [18]. In this study, a biophysical model was used to predict dispersal of larval propagules in the Irish Sea for *M*. *modiolus* reefs, followed by the use of microsatellite markers to directly test connectivity and validate the model to understand whether the genetic data had congruence with the modelling data. This study shows the applicability of using such genetic analysis in the context of protected habitats and is the first study to develop microsatellite markers of *M*. *modiolus*.

### Genetic Diversity and Particle Dispersal

The expected heterozygosity (H_e_) was highest within the Strangford Lough population and lowest in the Point of Ayre population suggesting greater gene diversity within the Strangford Lough population. The inbreeding co-efficient F_is_ was positive and significant for all populations and FreeNA analysis, reported null alleles for all loci at all populations (and zero for Modimicro 20). Overall, low genetic differentiation (and therefore more gene flow [18]) was recorded between the Ards Peninsula/Strangford Lough and North Llŷn/Point of Ayre populations.

Heterozygosity is an important measurement for population diversity [49]. A significant heterozygote deficiency has previously been observed in a number of marine bivalve species [50]. High mutation rate [51],the Walhund effect, inbreeding, non-panmixia or genotyping errors (null alleles; although not evident for all loci and populations herein and other scoring errors) [52] and selection may contribute to this phenomenon. Microsatellites in molluscs are known to be particularly susceptible to heterozygote deficiency due to technical artefacts, e.g. amplification failure during PCR (incomplete DNA purification or null alleles), incorrect genotyping or sampling drift due to small sample sizes [50]. Within this study, the highest number of alleles was reported within the Strangford Lough population, suggesting the richest genetic diversity of the 4 populations. However, a high degree of deviation for the HWE within this population, as well as the other 3 was noted, confirming heterozygote deficiency. High F_is_ values may indicate high levels of inbreeding, however, this is considered unlikely in bivalves with large populations and pelagic larvae [53]. It is likely that the high F_is_ values reported are due to the observed heterozygote deficiency and potentially to the quality of the sample DNA (DNA extraction for 3 of the 4 samples was undertaken at least 20 months after tissue storage in ethanol), however DNA quality was assessed prior to screening, with no apparent degradation of samples.

The results show that there is limited structure within the populations screened and that there is an overall weak differentiation of the Ards Peninsula/Strangford Lough and North Llŷn/Point of Ayre reefs. This would suggest that there is little restriction in gene flow between these two groups of populations.

Further development is required regarding the type of connectivity screening undertaken on priority marine organisms (in relation to informing policy and marine spatial planning). For example, it would be beneficial to screen potentially isolated populations or geographically distinct populations (e.g. east Scotland, Norway and Pacific e.g. [54]) to confirm applicability for microsatellite use within these populations. Within this study, microsatellites were identified as the genetic tool of choice based on the available resources at the time of study. Interpretation of the results of this study is limited by the low numbers of markers employed and the presence of null alleles; and further development of markers will be required to determine the full extent of the genetic connectivity of *M*. *modiolus* in the Irish Sea.

Results from the particle dispersal model show that larval settlement location is driven by oceanographic-atmospheric processes and larval duration. Simulations for passive larvae suggest that larval dispersal has the potential for dispersal to be wide reaching. This result corroborates the genetic analysis showing an inter-connected Irish Sea metapopulation. However, simulated populations on the east and west of the Irish Sea seem to be separate from one another, for several releases over a typical year due to variations in wind speeds and thermal heating [27]. The simulated large variability in dispersal (from one release date to another) allows us to speculate that, under different atmospheric conditions, such as greater thermal heating (resulting in strengthening of the residual frontal circulations), or persistent across-sea winds e.g. [55], it is possible that the Irish Sea populations may be more inter-connected However, all but the Strangford Lough population have been discovered in the last 20 years and the Ards population in the last 5 years, so, it is highly likely that other stepping-stone populations exist between those in the present study, increasing the likelihood of a connected network.

Combining the larval dispersal model and the genetic analysis results allows for the validation of the two methods and provides a more accurate depiction of the relationships between the populations. Discrepancies noted between the two methods in this study showed that, from a modelled perspective, the North Llŷn and Point of Ayre populations were not connected, whereas the genetic analysis showed no significant differentiation. Given the existence of a *M*. *modiolus* reef off the south of the Isle of Man (Little Ness) between Point of Ayre and North Llŷn, this site may contribute to the connectivity of these two populations if included in the larval dispersal model and could represent a stepping stone population.

Larval migration has been shown to be equally as influential to dispersal and connectivity as oceanographic controls [27]. However, sensitivity to larval duration has been investigated in the western Irish Sea gyre by Phelps et al. (2015)–resulting in relatively small influence with respect to larval connectivity and retention. Therefore, our results represent a sound baseline study in this area, and we are confident that natural variability in larval duration would not alter the general patterns of connectivity and retention presented here. Nevertheless, further work is required to provide more accurate larval migration, duration, and settlement data, as previously mentioned. In particular, the vertical swimming capabilities of *M*. *modiolus* are poorly understood—research suggests vertical migration that is synchronised with flood-tidal currents promotes coastal retention of larvae but also promotes long-distance dispersal to distant populations [27, 56]. On the other hand, larval migration that is synchronised with photoperiod does not promote dispersal to distant populations [27, 57]. Therefore, establishing accurate vertical larval behaviour for *M*. *modiolus* is vital before further biophysical modelling takes place. In addition, multi-year hydrodynamic modelling is required to quantify the natural intra-seasonal, inter-annual and climatic variability in physical controls on larval dispersal. Of particular influence will be periods with extreme events such as storms (producing both large waves and strong winds), which may increase dispersal in the upper water column, but may also break down residual pathways. Conversely, ‘heat waves’/droughts may strengthen residual pathways. Additionally, the influence of atypical conditions such as prolonged easterly winds should be quantified with regard to dispersal. Such information is crucial for improved model simulations of larval dispersal and connectivity.

### Management Implications

Due to the connectivity of the *M*. *modiolus* reefs implicated in this study and their decline and scarcity in the Irish Sea, it seems likely that they exist as a network of “stepping stone” habitats, each with an important role in gene flow. It was not possible to analyse Isolation by Distance of the populations in this study due to the limited number of markers developed but it would be useful to do so in the future, to understand in more detail the specific connections between each reef and identify direction of genetic flow (source/sink) to complement modelling outputs. Other known reefs are also scattered more widely along the west of the UK, perhaps indicating similar "stepping stones" for genetic exchange over the wider sea area.

Habitat connectivity (including genetics) of marine organisms has been cited as a major concern to the maintenance of marine biodiversity but despite the large number of published articles that assert the importance of using genetic data for management and conservation, very few use data applicable to real-life situations [58] as demonstrated in this study. An implication that arises from the notion of connected networks of protected habitats and species is the potential for the development of barriers to gene flow and the potential for genetic shift. Alteration of local hydrodynamics could result in disruption to the dispersal of larvae (and therefore genetic material). Careful management consideration is therefore required to ensure that connectivity between sites is maintained: in the design and selection of MPAs; in the placement of offshore developments (such as wind farms and tidal/wave devices); in conservation/biodiversity restoration programmes; and in the selection of appropriate management options in protected and wider sea areas (including fisheries management, invasive monitoring, and maintaining connectivity corridors).

The global literature on the connectivity of MPAs tends to be directed towards their benefits for commercial fish and the role of no-take reserves in increasing stocks in neighbouring areas and maintaining gene flow within populations [59–61]. However, little thought has been given to the connectivity of habitats, especially those that these fish inhabit in MPAs. Certain benthic habitats can be important to commercially valuable fish species [62, 63] and in some cases it is reported that juvenile fish have a greater chance of survival in structurally complex habitats [64]. This is relevant in the present study, because there is emerging evidence that structurally complex PMHs, including horse mussel reefs, are commercially important Essential Fish Habitat [65, 66].

The connectivity of PMHs may be of importance to commercial fisheries as well as to the biodiversity of an MPA network. Loss or damage to one of the stepping-stone habitats could have a knock-on effect to other areas of the marine ecosystem as a whole, resulting in consequences for biodiversity conservation and livelihoods, but also, in the achievement of objectives under MSFD, as the policy link has been made between the maintenance of PMHs and the achievement of GES [11].

It is well understood that *M*. *modiolus* reefs (and other benthic habitats) are particularly susceptible to damage from certain types of fishing gear [1, 3, 6, 67, 68]. In Strangford Lough >10km^2^ of *M*. *modiolus* reefs have been lost since the mid-1970s [13, 69] and were associated with high levels of biodiversity [13, 69]. Currently, efforts are underway to restore the Strangford Lough *M*. *modiolus* reefs with recent considerations including the transfer of adult mussels to areas characterized as having high potential for successful translocation (i.e. according to habitat suitability, larval dispersal modelling and historical distribution of the species) and falling within a proposed non-disturbance zone [13, 70]. However, given that translocation requires acquiring sufficient number of *M*. *modiolus* from outside the area, the issue of introducing genetically non-compatible animals has been raised [13]. Genetic similarity shown in this study supports the proposal that the Ards Peninsula *M*. *modiolus* population may be a prospective source of *M*. *modiolus* for translocation to Strangford Lough [13, 70, 71]. On the other hand, the present study also indicates that, should appropriate ground be available in Strangford Lough, larvae from the Ards Peninsula are also capable of reaching it and, given the right conditions, to colonise it.

Overall, in the Irish Sea, the general decline in horse mussel beds, and the apparent connectivity of the remaining beds (as seen from larval dispersal and genetic similarity in this study), implies that it would be wise to manage them as inter-dependent stepping-stones, especially on either side of the Irish Sea. Creating a coherent network of MPAs and the achievement of GES the MSFD by 2020, requires information on the connectivity of populations; research on which is currently lacking across Europe. Greater study resolution and research over a larger spatial area will improve the ability to design MPA networks and could further inform future management; therefore achieving long term protection of Europe’s marine environment.
